# Demographic Effects on Longitudinal Semantic Processing, Working Memory, and Cognitive Speed

**DOI:** 10.1093/geronb/gbaa080

**Published:** 2020-07-01

**Authors:** Jet M J Vonk, Eve Higby, Alexandre Nikolaev, Dalia Cahana-Amitay, Avron Spiro, Martin L Albert, Loraine K Obler

**Affiliations:** 1 Taub Institute for Research on Alzheimer’s Disease and the Aging Brain, Department of Neurology, Columbia University Irving Medical Center, New York, New York; 2 Julius Center for Health Sciences and Primary Care, University Medical Center Utrecht, The Netherlands; 3 Department of Speech, Language, and Hearing Sciences, California State University, East Bay, Hayward; 4 Department of Psychology, University of California, Riverside; 5 Helsinki Collegium for Advanced Studies, University of Helsinki, Finland; 6 6School of Languages and Cultures, University of Sheffield, Massachusetts; 7 Department of Neurology, Boston University School of Medicine, Massachusetts; 8 Veterans Affairs Boston Healthcare System, Massachusetts; 9 Department of Epidemiology, School of Public Health, Boston University, Massachusetts; 10 The Graduate Center of the City University of New York

**Keywords:** Aging, Cognition, Factor analysis, Language, Trajectory

## Abstract

**Objectives:**

To better understand and compare effects of aging and education across domains of language and cognition, we investigated whether (a) these domains show different associations with age and education, (b) these domains show similar patterns of age-related change over time, and (c) education moderates the rate of decline in these domains.

**Method:**

We analyzed data from 306 older adults aged 55–85 at baseline of whom 116 returned for follow-up 4–8 years later. An exploratory factor analysis identified domains of language and cognition across a range of tasks. A confirmatory factor analysis analyzed cross-sectional associations of age and education with these domains. Subsequently, mixed linear models analyzed longitudinal change as a function of age and moderation by education.

**Results:**

We identified 2 language domains, that is, semantic control and semantic memory efficiency, and 2 cognitive domains, that is, working memory and cognitive speed. Older age negatively affected all domains except semantic memory efficiency, and higher education positively affected all domains except cognitive speed at baseline. In language domains, a steeper age-related decline was observed after age 73–74 compared to younger ages, while cognition declined linearly with age. Greater educational attainment did not protect the rate of decline over time in any domain.

**Discussion:**

Separate domains show varying effects of age and education at baseline, language versus cognitive domains show dissimilar patterns of age-related change over time, and education does not moderate the rate of decline in these domains. These findings broaden our understanding of age effects on cognitive and language abilities by placing observed age differences in context.

Aging is generally accompanied by increased cognitive difficulties (e.g., Reuter-Lorenz and Park, 2014; Salthouse, 2010; Schaie, 2005; Verhaeghen and Salthouse, 1997), but the level of performance across older age can vary substantially from one individual to another. One factor that has been consistently reported to influence cognitive abilities in aging is educational attainment; a history of higher education is associated with better preserved cognitive abilities in older age (e.g., Albert et al., 1995; Manly et al., 1999; Meara et al., 2008). Positive influence of education on cognitive performance across older adulthood has been demonstrated in various domains, including memory, processing speed, reasoning, working memory, and executive functions such as inhibition, shifting, and abstraction (Van der Elst et al., 2006; Van Hooren et al., 2007). While education moderates the level of cognitive performance in older adults, longitudinal studies of cognitive decline have shown no effect of education on the rate of change over time (e.g., Christensen et al., 2001, 2009; Der et al., 2010; Seeman et al., 2005; Tucker-Drob et al., 2009; Van Dijk et al., 2008; Zahodne et al., 2011).

As with cognitive abilities, certain language abilities also tend to decline in older adulthood (e.g., Kempler et al., 1998) and education positively influences language task performance in older adults (e.g., Constantinidou et al., 2012; Goral et al., 2011; Seeman et al., 2005; Verhaeghen, 2003). However, little is known about the influence of education on different types of language processing skills and change in performance across older age (Kempler et al., 1998). One of the few studies on this topic showed that the decline in lexical retrieval tasks became more rapid with older age and that age-related change was larger for individuals with lower education (Goral et al., 2007). More research is needed to examine the effects of education on different domains of language and to separate the effects of age and education on the language versus cognitive components in linguistic tasks.

Different domains of language are rarely compared in the aging literature to cognitive subdomains such as executive functioning and processing speed. Language depends on and is intertwined with many other cognitive systems, such as attention, decision making, and memory (e.g., Meier et al., 2016). Multiple theoretical perspectives also acknowledge that there are—to a greater or lesser extent—differences between language and other aspects of cognition (e.g., Cahana-Amitay and Albert, 2014; Harris, 2003). Distinctions between language and cognitive processing, as well as among various aspects of language processing, become more apparent in aging (Rastle and Burke, 1996). Several applications of the fluid/crystallized intelligence model (i.e., the Cattell–Horn–Carroll theory of cognitive abilities; Flanagan and Dixon, 2013) have shown that cognitive skills such as reasoning, spatial visualization, memory, and speed (i.e., fluid intelligence) decline linearly with age, while vocabulary knowledge (i.e., crystalized intelligence) improves across the life span (Horn and Cattell, 1966, 1967; Salthouse, 2010). Innately, crystalized intelligence closely relates to various language abilities, including vocabulary, reading comprehension, and conversational fluency.

However, evidence from several experimental studies of age-related decline in semantic memory (e.g., Bowles and Poon, 1985; Byrd, 1984), which includes vocabulary, have instigated an ongoing discussion on different components involved in semantic memory functioning and their differential relations with aging (e.g., Bäckman and Nilsson, 1996; Gordon et al., 2018; Pistono et al., 2019). For example, the Transmission Deficit Hypothesis (Burke et al., 1991) proposes that word retrieval difficulties in so-called “tip-of-the-tongue” experiences are caused by adequate retrieval of a concept from semantic memory, but a failure to connect to all phonological nodes to form its name at the phonological level—this latter process being particularly vulnerable in aging. As well, various studies have highlighted the effect of cognitive functions, such as processing speed and executive function, on semantic memory functioning (e.g., Cansino et al., 2020; Spaan, 2015). Among the different components of semantic memory functioning that have been identified are *semantic control*, that is, the ability to use semantic and grammatical information in the relevant context (Badre and Wagner, 2007; Cahana-Amitay et al., 2016; Chiou et al., 2018; Hoffman, 2018; Jefferies and Lambon Ralph, 2006; Whitney et al., 2011), and *semantic memory efficiency*, that is, the ability to access information quickly from long-term memory (Adrover-Roig et al., 2012; Fisk and Sharp, 2004; Higby et al., 2019).

This study aimed to refine our understanding of the effects of older age and education across domains of language and cognition and their effects on change over time. By using a data-driven approach to identify domains of language and cognition across a range of linguistic and cognitive tasks, we sought to investigate whether (a) these domains show different associations with age and education, (b) these domains show similar patterns of age-related change over time, and (c) education moderates the rate of change in these domains.

## Method

### Participants

We included 306 healthy, community-dwelling adults aged 55–85 at baseline from the Language in the Aging Brain project, a prospective cohort designed to investigate the relations between cognition and language in aging and the influence of health on these relations (Cahana-Amitay et al., 2016; Higby et al., 2019). Participant characteristics are presented in Table 1. Recruitment was described in detail elsewhere (Higby et al., 2019). All participants used English as their primary language and learned English before age 7. Education ranged from 9 to 17+ years and was divided into three categories: high-school graduation or less (≤12 years), college (13–16 years), or advanced (masters/doctoral) degree; >16 years).

**Table 1.** Participant Characteristics (*n* = 306)

Age at baseline, mean (*SD*; range)	71.60 (7.69; 55–84)
Sex/gender, *n* (% women)	150 (49.0)
Education, *n* (%)	
High school	59 (19.3)
College	155 (50.7)
Masters/doctoral	92 (30.1)
Race/ethnicity, *n* (%)	
Non-Hispanic White	246 (84.5)
Non-Hispanic Black	40 (13.7)
Hispanic	2 (0.7)
Other	3 (1.0)
MMSE, mean (*SD*; range)	28.91 (1.20; 24–30)

*Note:* MMSE = Mini-Mental State Examination.

Of the participants, 116 returned for follow-up testing 4–8 years later (mean = 6.6 years, range 4.2–8.8 years). We tested for measurement invariance over time to ensure that the internal structure of our measurement battery was equal across time (e.g., Avila et al., 2020; see Supplementary Text 3).

Individuals were excluded from testing if they had a history of stroke, head trauma, neurodegenerative or significant psychological disorders, and/or if they had had intensive medical treatment (e.g., dialysis, chemotherapy) within 1 year of testing (depending on the specific treatment). Individuals were asked to bring glasses and hearing aids if applicable, and hearing acuity was verified through audiometric assessment. All participants gave written consent in accordance with the Institutional Review Boards of the Boston University School of Medicine and Veterans Affairs Boston Healthcare System.

### Cognitive and Language Assessment

To assess different aspects of language processing, our test battery included noun and verb confrontation naming (Boston Naming Test [BNT] and Action Naming Test [ANT]), sentence processing (Embedded Sentences Task and Multiple Negatives Task), and verbal fluency (letter and animal fluency). Cognition was assessed with tasks of working memory (Month Ordering and Digit Ordering), executive functions (shifting: Trail-making Test; inhibition: Stroop Test), and cognitive speed (Letter Comparison and Pattern Comparison). Detailed descriptions of the tasks are available in Supplementary Text 1. Mean task scores at baseline and follow-up are provided in Table 2.

**Table 2.** Test Scores at Baseline and Follow-up

	Baseline	Follow-up
ANT accuracy, mean (*SD*; range)	95.98 (3.88; 94.64–98.23)	95.25 (4.48; 92.97–98.23)
ANT RT, mean (*SD*; range)	1353.74 (310.18; 1137.94–1512.85)	1465.20 (295.63; 1267.66–1615.72)
BNT accuracy, mean (*SD*; range)	92.62 (6.83; 89.73–98.22)	91.55 (8.96; 89.16–98.33)
BNT RT, mean (*SD*; range)	1296.22 (273.07; 1099.62–1430.03)	1386.34 (326.22; 1145.28–1590.40)
Embedded Sentences Task, mean (*SD*; range)	89.18 (8.62; 84.72–94.44)	84.92 (13.71; 81.25–93.06)
Multiple Negatives Task, mean (*SD*; range)	92.60 (6.22; 90.00–96.00)	91.79 (7.51; 88.00–98.00)
Letter Fluency, mean (*SD*; range)	45.76 (13.60; 37.00–55.00)	47.24 (14.85; 39.00–57.00)
Animal Fluency, mean (*SD*; range)	17.55 (5.24; 14.00–21.00)	17.62 (6.61; 14.00–21.00)
Month Ordering span, mean (*SD*; range)	4.27 (0.99; 3.50–5.00)	4.44 (0.85; 4.00–5.00)
Digit Ordering span, mean (*SD*; range)	4.61 (0.87; 4.00–5.00)	4.07 (0.79; 3.50–5.00)
Stroop difference score, mean (*SD*; range)	147.90 (40.67; 124.00–173.00)	151.74 (42.71; 131.50–170.00)
Trails difference score, mean (*SD*; range)	47.20 (30.05; 27.00–59.00)	50.05 (36.00; 26.00–64.25)
Letter Comparison, mean (*SD*; range)	17.06 (4.23; 14.00–20.00)	15.78 (4.09; 12.00–19.00)
Pattern Comparison, mean (*SD*; range)	28.66 (5.93; 25.00–32.00)	27.95 (5.98; 24.00–32.00)

*Note*: ANT = Action Naming Test; BNT = Boston Naming Test; RT = response time.

### Statistical Analysis

A detailed description of the statistical analyses is presented in Supplementary Text 2. In short, participant characteristics were analyzed using descriptive statistics, chi-squared tests, and general linear models. We performed multiple imputation to account for missing data on education for 14 participants (4.6%). A comparison of the characteristics of participants with and without education data, as well as a comparison of returners versus non-returners on demographic variables and test variables at baseline, is provided in Supplementary Table S1. Due to skewness, scores on the Trails task and accuracy for ANT, BNT, Embedded Sentences Task, and Multiple Negatives Task were transformed; additionally, scores were transformed such that on every test a higher score reflected better performance (details on transformations are provided in Supplementary Text 2).

To model the underlying factor structure of the cognitive and language tasks, we performed an exploratory factor analysis (EFA) obtaining an eigenvalue analysis, including a scree plot. We followed standard guidelines for goodness of fit (i.e., Root Mean Square Error of Approximation [RMSEA], Comparative Fit Index [CFI], Tucker Lewis Index [TLI], and Standardized Root Mean Squared Residual [SRMR]; Yu, 2002), and used Akaike Information Criterion (AIC) for model comparison. Item loadings with values of 0.25 or larger were considered for each factor. 

We performed confirmatory factor analysis (CFA) to investigate if the effects of age and education were different among the latent factors identified by the EFA. The CFA model included the identified factors from the EFA as latent variables based on the observed cognitive and language tasks at baseline, with age, education, and sex regressed on the latent variables. After model specification, model fit was assessed using RMSEA, CFI, TLI, and SRMR. Modification indices combined with conceptual judgment were used for model improvement, and model improvement was assessed by AIC values. The resulting model was used for hypothesis testing. To compare the estimates of the effects of age and education on the latent factor parameters, linear restrictions on the parameters in the model were tested using the Wald chi-squared test.

Change over time in the latent factors defined in the CFA was investigated using linear mixed models. Mixed models included the latent factors as the dependent variables. Time in the study parameterized by age (from baseline age to follow-up age, which accounts for individually varying follow-up intervals), as well as educational attainment and sex, were included as fixed factors, together with a random intercept and random slope. Subsequent models additionally included the interaction between time in study (parameterized by age) and educational attainment to test for moderation by education on slope. A basis spline was fitted to perform piecewise linear modeling within the mixed model. AICs of models without and with a spline were compared, and optimal placement of knots was assessed by comparing models’ AIC.

Multiple imputation was performed in SPSS version 25, EFA and CFA were analyzed in Mplus version 8, and participant characteristics, linear mixed models, and visualization were performed in R version 3.6.0.

## Results

### Exploratory Factor Analysis

The correlation matrix of the 14 language and cognitive tasks at baseline is presented in Supplementary Table S2. Following Evans (1996), the strength of correlations can be described ranging from very weak (.00–.19), weak (.20–.39), moderate (.40–.59), strong (.60–.79), to very strong (.80–1.0). Inspection of the correlation matrix indicated that the Stroop task only had very weak correlations with the other tasks. Initial factor analyses that included the Stroop task showed that this task did not load sufficiently onto any of the factors. Because a factor analysis determines the underlying dimensions across tasks based on correlations and covariances, we excluded this task from further analyses and ran the final EFA with the remaining 13 tasks.

Factor loadings and model fit information are presented in Table 3. An eigenvalue analysis yielded three values above one, 1: 4.408 (the cumulative percentage of variability explained 33.9%), 2: 1.574 (46.0%), 3: 1.223 (55.4%), with the fourth value falling just below one, 4: 0.946 (62.7%), and subsequent values falling considerably below one. The scree plot leveled off after four factors.

**Table 3.** Exploratory Factor Analysis: Factor Loadings and Model Fit Information

# factors in model	1	2		3			4				5					6					
ANT acc	**0.576**	**0.401**	**0.285**	**0.545**	**0.370**	0.019	**0.657**	0.135	−0.033	0.019	**0.775**	0.022	−0.059	0.024	0.003	−0.019	**0.726**	−0.082	0.095	0.009	−0.017
ANT RT	**0.530**	**0.800**	−0.096	−0.038	**0.592**	**0.569**	0.068	**0.672**	−0.039	0.077	0.064	**0.710**	−0.026	0.052	−0.064	**10.607**	0.005	−0.007	0.005	−0.003	0.000
BNT acc	**0.577**	**0.467**	0.225	**0.562**	**0.490**	−0.020	**0.655**	**0.262**	−0.011	−0.116	**0.601**	0.226	0.033	−0.096	0.003	0.007	**0.793**	0.017	0.007	−0.119	−0.001
BNT RT	**0.604**	**0.806**	0.001	0.063	**0.557**	**0.560**	−0.018	**0.862**	0.099	0.012	0.022	**0.814**	−0.033	0.026	0.072	0.161	**0.474**	0.161	**−0.256**	0.160	0.019
EST	**0.509**	0.077	**0.545**	**0.588**	−0.039	−0.001	**0.432**	−0.160	0.181	0.223	**0.323**	−0.060	**0.458**	0.127	−0.036	−0.004	0.110	0.049	**0.567**	0.193	0.008
MNJ	**0.366**	−0.012	**0.476**	**0.554**	−0.061	−0.130	**0.290**	−0.117	**0.294**	0.026	0.192	−0.057	**0.358**	−0.011	0.120	0.050	0.022	−0.007	**0.439**	0.003	0.202
Letter-F	**0.539**	0.209	**0.444**	**0.381**	−0.001	0.235	0.217	0.024	0.171	**0.297**	0.188	0.061	0.170	**0.247**	0.124	−0.048	0.206	0.044	0.065	**0.300**	0.143
Animal-F	**0.603**	**0.368**	**0.384**	**0.382**	0.173	**0.266**	0.154	**0.290**	**0.286**	0.107	−0.012	**0.478**	**0.499**	−0.027	0.033	−0.011	0.011	**10.110**	0.016	−0.001	0.010
MO	**0.497**	−0.045	**0.722**	**0.644**	−0.205	0.007	0.006	−0.008	**0.848**	−0.019	−0.009	0.017	**0.383**	0.006	**0.548**	0.028	−0.056	0.063	0.213	0.006	**0.698**
DO	**0.397**	−0.012	**0.549**	**0.482**	−0.155	0.037	−0.044	0.075	**0.617**	−0.009	0.001	−0.012	−0.007	0.016	**0.747**	−0.032	0.083	−0.041	−0.081	0.002	**0.695**
Trails	**0.567**	0.226	**0.467**	**0.408**	0.023	0.235	0.209	0.058	**0.244**	**0.248**	0.191	0.092	0.145	0.193	0.205	0.013	**0.223**	0.016	0.039	**0.229**	**0.249**
LC	**0.599**	**0.323**	**0.402**	0.021	−0.146	**0.792**	−0.025	−0.004	0.032	**0.851**	0.018	−0.022	−0.049	**0.977**	0.066	−0.013	−0.017	−0.081	−0.005	**0.929**	0.039
PC	**0.512**	**0.369**	0.242	−0.111	−0.021	**0.772**	0.002	0.068	−0.099	**0.714**	−0.039	0.223	0.143	**0.536**	−0.117	0.043	0.008	0.078	0.001	**0.680**	−0.117
χ ^2^ Model Fit	323.59, df = 65, *p* < 0.001	168.10, df = 53, *p* < 0.001		87.40, df = 42, *p* < 0.001			36.51, df = 32, *p* = 0.267				19.47, df = 23, *p* = 0.674					5.34, df = 15, *p* = 0.989					
RMSEA	0.114	0.084		0.059			0.021				< 0.001					<0.001					
CFI	0.693	0.863		0.946			0.995				1.000					1.000					
TFI	0.632	0.799		0.900			0.987				1.014					1.060					
SRMR	0.086	0.057		0.037			0.025				0.018					0.010					
AIC	18749.21	18617.71		18558.99			18528.12				18529.09					18530.95					

*Note:* acc = accuracy; Animal-F = Animal Fluency; ANT = Action Naming Test; BNT = Boston Naming Test; DO = Digit Ordering span; EST = Embedded Sentences Task; LC = Letter Comparison; Letter-F = Letter Fluency; MNT = Multiple Negatives Task; MO = Month Ordering span; PC = Pattern Comparison; RT = response time. A seven-factor model did not converge. Bold indicates loadings >0.25 and loadings <0.25, and those in which a test loaded more strongly on another factor (difference between absolute factor loadings >0.05) are not highlighted.

The chi-squared Goodness of Fit test compares the observed sample distribution with the expected probability distribution, which should not differ from each other; based on this test, the models with one, two, and three factors were rejected (*p* < .05) but models with four, five, and six factors were not (*p* > .05). The AIC was smallest for the four-factor model. The RMSEA, CFI, TFI, and SRMR indices all indicated that the four-factor model fit the data well. Therefore, we considered the four-factor model to best describe the underlying dimensions of the cognitive and language variables, and we used this model in subsequent analyses.

We considered the strongest item loading for each task (with values of ≥0.25) or multiple loadings if a task loaded equally strong on more than one factor (i.e., if the difference between absolute factor loadings <0.05). Tasks loading onto the first factor were ANT accuracy, BNT accuracy, the Embedded Sentences Task, and the Multiple Negatives Task. Therefore, we labeled this factor *semantic control*. The second factor included loadings for ANT response time, BNT response time, and Animal Fluency; this factor is thought to represent *semantic memory efficiency* Note that response time means were calculated on accurate trials only and therefore reflect successful semantic processing (see Supplementary Text 1). Tasks loading onto the third factor were the Multiple Negatives Task, Animal Fluency, Month Ordering, Digit Ordering, and Trails, which we labeled *working memory*. Lastly, the fourth factor included loadings for Letter Fluency, Trails, Letter Comparison, and Pattern Comparison, and was therefore considered to represent *cognitive speed*. The Multiple Negatives Task, Animal Fluency, and Trails each loaded more or less equally onto two latent factors (with a difference between absolute factor loadings of <0.05).

Factor correlations are presented in Table 4. Correlations among factors ranged from 0.134 (semantic memory efficiency and working memory) to 0.443 (working memory and cognitive speed). All tasks had at least a moderate correlation with the factor they loaded onto. Additionally, certain tasks correlated with other factors as well, reflecting the multicomponent nature of certain tasks, such as Animal Fluency (Shao et al., 2014).

**Table 4.** Confirmatory Factor Analysis: Correlations Among Factors in the Four-Factor Model

Factors	Semantic control	Semantic memory efficiency	Working memory	Cognitive speed
Semantic control	1			
Semantic memory efficiency	.404	1		
Working memory	.410	.134	1	
Cognitive speed	.347	.419	.443	1

### Confirmatory Factor Analysis

The CFA included four latent factors reflecting semantic control, semantic memory efficiency, working memory, and cognitive speed. Model fit of this CFA, with age, education, and sex regressed on the latent variables, did not meet thresholds for most of the recommended fit values: RMSEA = .073, CFI = .865, TLI = .810, and SRMR = .071. The AIC of this model was 8726.86.

**Figure 1.**
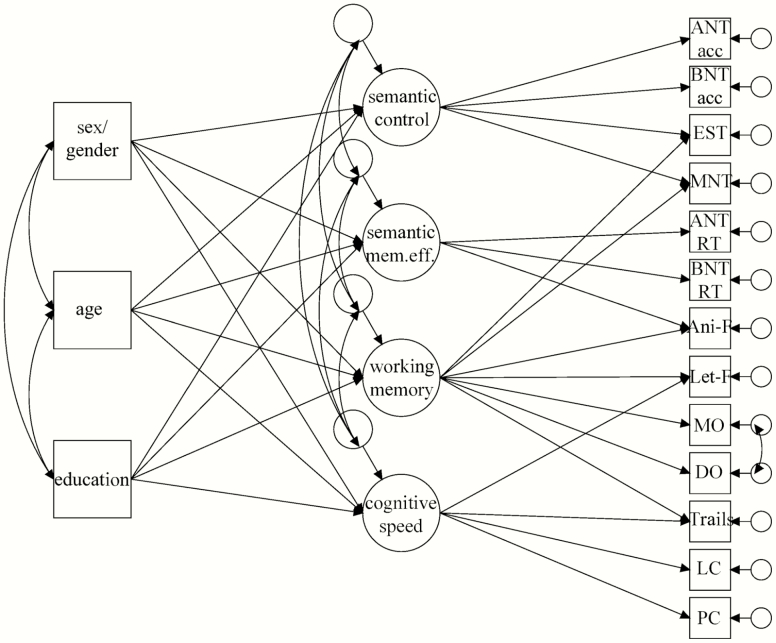
Final confirmatory factor analysis model. *Note*: Acc = accuracy; ani-F = Animal Fluency; ANT = Action Naming Test; BNT = Boston Naming Test; DO = Digit Ordering span; EST = Embedded Sentences Task; let-F = Letter Fluency; LC = Letter Comparison; MNT = Multiple Negatives Task; MO = Month Ordering span; PC = Pattern Comparison; RT = response time; semantic mem. eff. = semantic memory efficiency.

Modification indices suggested that the Embedded Sentences Task and Letter Fluency load onto working memory as well; we considered these modifications appropriate as the Embedded Sentences Task, similar to the Multiple Negatives Task, and the Letter Fluency task, like Animal Fluency, are multicomponent tasks that rely on working memory abilities as well. Addition of these paths in the model improved model fit (Embedded Sentence: RMSEA = .065, CFI = .894, TLI = .848, and SRMR = .059, AIC = 8699.27; Letter Fluency: RMSEA = .062, CFI = .905, TLI = .862, and SRMR = .054, AIC = 8689.21). Additionally, modification indices suggested allowing the measurement errors of Month Ordering and Digit Ordering to correlate; we considered this modification theoretically appropriate as these tasks are quite similar. Addition of this path in the model improved model fit to the recommended standards: RMSEA = .051, CFI = .936, TLI = .907, and SRMR = .048, with the lowest AIC value of 8658.55 compared to the previous models. No other modification indices for model improvement were theoretically valid. The final model is shown in Figure 1, and all factor loadings, effects on latent variables, and covariances are reported in Supplementary Table S3.

Age was negatively related to semantic control (*B* = −.012, *SE* = 0.006, *p* = .042), working memory (*B* = −.022, *SE* = 0.006, *p* = .001), and cognitive speed (*B* = −.011, *SE* = 0.005, *p* = .020), but not to semantic memory efficiency (*B* = −.007, *SE* = 0.007, *p* = .304). The Wald chi-squared test to compare the estimates of the effects of age and education on the latent factor parameters showed that the magnitude of the effect of age did not differ between any of the latent factors: semantic control versus semantic memory efficiency (*p* = .431), semantic control versus working memory (*p* = .140), semantic control versus cognitive speed (*p*= .880), semantic memory efficiency versus working memory (*p* = .065), semantic memory efficiency versus cognitive speed (*p* = .567), or working memory versus cognitive speed (*p* = .122).

Higher educational attainment was related to higher semantic control (*B* = .342, *SE* = 0.069, *p* < .001), semantic memory efficiency (*B* = .208, *SE* = 0.071, *p* = .003), and working memory (*B* = .252, *SE* = 0.078, *p* = .001), but not cognitive speed (*B* = .064, *SE* = 0.039, *p* = .104). The Wald chi-squared test showed the effect of education was smaller on cognitive speed than semantic control (*p* < .001), semantic memory efficiency (*p* = .050), and working memory (*p* = .028). The effect of education did not differ between semantic control and semantic memory efficiency (*p* = .084), semantic control and working memory (*p* = .290), or semantic memory efficiency and working memory (*p* = .654).

### Longitudinal Change

**Figure 2.**
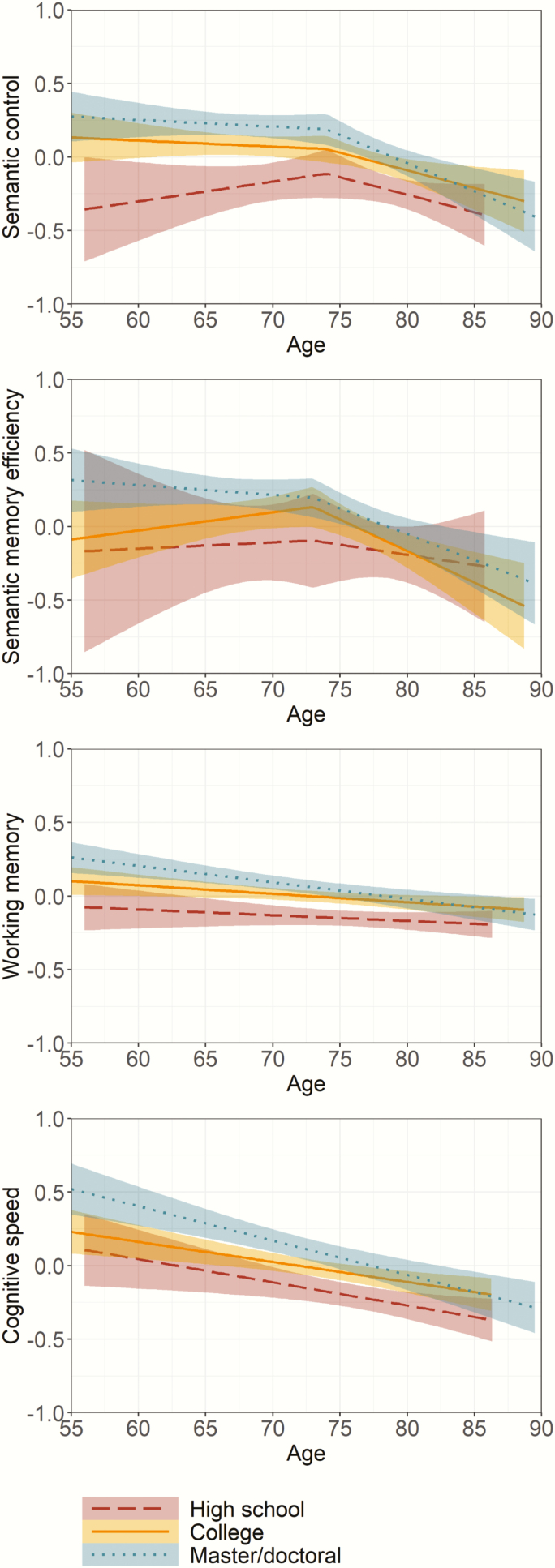
Change over two assessments across educational levels as a function of age.

Change over two assessments by educational level as a function of age, adjusted for sex, is shown in Figure 2 for each of the four latent cognitive domain scores. A loess curve fitted on scores over time for semantic control implied little decline at younger ages followed by increasing decline. In the model, a basis spline was fitted between 70 and 80 years; comparison of AIC values confirmed better model fit with a spline and indicated best model fit with a knot at 74 years of age. For individuals 55–74 years of age, semantic control did not decline over time (*B* = −.045, *SE* = 0.068, *p* = .505), but for individuals older than 74 semantic control declined with increasing age (*B* = −.552, *SE* = 0.082, *p* < .001). Educational attainment influenced initial level of semantic control, such that those with the highest education had higher semantic control than those with some college (*B* = .124, *SE* = 0.047, *p* = .009) or high school (*B* = .297, *SE* = 0.062, *p* < .001), and those with some college had higher semantic control than those with high school education (*B* = .173, *SE* = 0.057, *p* = .003). Education moderated the rate of change after 74 years of age such that the slope of those with only high school was less steep than those with some college (*B* = .473, *SE* = 0.236, *p* = .048) or advanced education (*B* = .630, *SE* = 0.245, *p* = .012).

For semantic memory efficiency, the loess curve also suggested no decline at younger ages followed by increasing decline. A basis spline was fitted on ages between 70 and 80, and the AIC confirmed that a model with spline fit better than one without; model fit was best with a knot at 73 years of age. Semantic memory efficiency did not decline in individuals up to 73 years of age (*B* = .055, *SE* = 0.109, *p* = .618), but declined with age among older individuals (*B* = −.668, *SE* = 0.132, *p* < .001). Those with the highest education had higher semantic memory efficiency than those with high school (*B* = .254, *SE* = 0.091, *p* = .006) or college (*B* = .190, *SE* = 0.070, *p* = .007), but there was no difference between those with college or high school education (*B* = .064, *SE* = 0.085, *p* = .451). The slope of decline after 73 years of age was not moderated by educational attainment.

Working memory declined linearly with age (*B* = −.008, *SE* = 0.002, *p* < .001). Initial level of working memory was lower in those with high school compared to those with college (*B* = −.124, *SE* = 0.040, *p* = .002) or higher (*B* = −.176, *SE* = 0.044, *p* < .001). There was no difference in initial level of working memory between those with college or higher education (*B* = .052, *SE* = 0.034, *p* = .125). Educational attainment did not moderate the slope of decline.

Cognitive speed also declined linearly with age (*B* = −.018, *SE* = 0.003, *p* <. 001). Those with the highest education had faster cognitive speed than those with high school (*B* = .221, *SE* = 0.067, *p* = .001) or college (*B* = .102, *SE* = 0.051, *p* = .046). Cognitive speed between those with high school versus college was less distinguishable (*B* = .119, *SE* = 0.062, *p* = .056). The rate of decline in cognitive speed was not moderated by educational attainment.

## Discussion

This study aimed to better understand and compare the effects of aging and education across domains of language and cognition. We investigated whether (a) these domains show different effects of age and education, (b) these domains show similar patterns of age-related change over time, and (c) education moderates the rate of decline in these domains. Using both cross-sectional and longitudinal approaches, the results showed that aging affects language and cognitive domains differently in older adults between 55–85 years of age. Education, in contrast, has a common effect across language and cognitive domains with more benefit in absolute scores for the higher educated across all older-adult ages, but no corresponding beneficial effect on the rate of decline over time. A factor analysis of the different language tasks used in this study, including object and action naming, two sentence processing tasks, and verbal fluency, in combination with several cognitive tasks, showed that different aspects of language load onto either semantic control or semantic memory efficiency; these results correspond to a large extent with the factor analysis of cognitive tasks by Adrover-Roig et al. (2012).

Our results support the proposal that semantic cognition encompasses multiple components, including the existence of the relatively underdiscussed but distinct concept of semantic control in cognitive aging (e.g., Chiou et al., 2018; Hoffman, 2018; Jefferies et al., 2007)—particularly in the context of change in linguistic abilities in older age (e.g., Cahana-Amitay et al., 2016). Semantic control reflects what aspects of a concept are retrieved from memory and what is done with that information based on task demands (e.g., Whitney et al., 2011). Semantic information is employed strategically through controlled processing of conceptual meaning and application of such knowledge in the appropriate and task-relevant context (Badre and Wagner, 2007; Cahana-Amitay et al., 2016; Jefferies and Lambon Ralph, 2006). Although still not well specified, single-word retrieval is thought to require control processes in order to resolve competition among lexical candidates, monitor selection processes, and self-prime when a word’s form is elusive (Adrover-Roig et al., 2012; Nozari and Novick, 2017; Shao et al., 2012). In sentence contexts, semantic information needs to be manipulated and integrated to form a coherent meaning and determine sentence plausibility. Semantic memory efficiency, by contrast, allows for effective access to the storage of conceptual meanings, including the categories that concepts belong to. Semantic information is thought to be organized in a network, in which semantic activation spreads through related concepts (e.g., Vonk, Flores, et al., 2019). Thus, a task like Animal Fluency reflects the ability to quickly and accurately access concepts related to a category like *animals*. Similarly, since the response times for ANT and BNT were based on accurate responses only, these naming speed measures reflect the efficiency of successful semantic processing as well.

The different cognitive requirements for semantic control versus semantic memory efficiency are also reflected in the correlations among the latent factors identified in this study: semantic control correlated with working memory, as both rely on controlled processing and manipulation of information, whereas semantic memory efficiency correlated with cognitive speed, as both contribute to efficiency of processing. This differential correlation could also explain the previously found effects of executive function on semantic memory functioning (e.g., Cansino et al., 2020; Spaan, 2015) as being particularly driven by the semantic control component of semantic memory. Importantly, in the cross-sectional analyses, age was not related to semantic memory efficiency, but it was related to semantic control and the two cognitive domains of working memory and cognitive speed. This differential association of age with semantic control but not semantic memory efficiency replicates the findings by Hoffman (2018), including his finding that an analysis of response times on two semantic memory tests—much like the inclusion of response times in our latent factor of semantic memory efficiency—showed no differences between age groups. The absence of a relation between semantic memory efficiency and age also fits within the fluid/crystalized intelligence model, in which semantic memory efficiency would be considered crystalized intelligence (closely linked to vocabulary), while semantic control, working memory, and cognitive speed would be considered fluid intelligence. The results are also in line with the Transmission Deficit Hypothesis (Burke et al., 1991), in which processes involved in connecting semantic memory to the lexical and phonological levels of word retrieval (which could be considered semantic control) are affected by aging.

Age is commonly a significant predictor of cognitive and language performance in studies of older adults (e.g., Harada et al., 2013; Reuter-Lorenz and Park, 2014; Salthouse, 2010; Schaie, 2005; Verhaeghen and Salthouse, 1997). In the current study, however, age appears to relate somewhat differently to performance on language and cognitive tasks. In particular, the results of the CFA showed that age did not affect semantic memory efficiency. The longitudinal analyses confirmed this finding up until 74 years, after which decline became apparent. Semantic control showed a similar longitudinal pattern, with no substantial change up until about 73 years of age, after which performance declined; however, the effects of age on semantic control were also present in cross-sectional analyses. This nonlinear pattern of change over time in semantic control and semantic memory efficiency, dependent on age, may have methodological implications for dividing older adults into groups in future studies. Consistent with well-established patterns in the literature, our results showed performance on working memory and cognitive speed to become worse with older age and to decline linearly over time (e.g., Brockmole and Logie, 2013; Salthouse, 1994, 2000; Zahodne et al., 2011). The pattern of age relating differently to language than cognition in change over time—with a steeper decline in language domains among older participants (starting at about 73–74 years of age) in comparison to the younger ones—was also observed in a meta-analysis by Feyereisen (1997). These results also follow the general direction of age-related changes described within the fluid/crystallized intelligence model (Horn and Cattell, 1967), and age-related changes that influence the model’s factor structure (Baltes and Kliegl, 1986).

The majority of recent longitudinal studies have reported no protective effect of education on age-related cognitive change within individuals (for a review, see Lenehan et al., 2015). Consistent with this large body of research, we found that educational attainment had a favorable effect on the initial level of language and cognitive performance in healthy older adults, with the exception of cognitive speed (e.g., Van Hooren et al., 2007), but generally did not affect the rate of decline over time (e.g., Vonk, Arce Rentería, et al., 2019; Zahodne et al., 2011). The one exception was semantic control: rather than having a protective effect for individuals with higher education, the slope of those with education beyond high school was steeper than that of individuals with less education. We suspect that this finding may be related to intercept−slope correlation, that is, those who start at a higher intercept have a greater dip in scores, resulting in a steeper slope (Lawrence et al., 2008; Silver et al., 2005). The absence of an effect of education on baseline cognitive speed is in line with findings by Ritchie et al. (2013) (but see Zahodne et al., 2011).

The lack of a protective effect of education may be due to measuring education in years of formal schooling. Years of education is the most often used proxy to represent “cognitive reserve,” which is the ability to maintain cognitive function in the face of neurodegenerative changes (e.g., Stern, 2002; Stern et al., 2018). The idea underlying cognitive reserve is that some aspect of older individuals’ cognitive abilities is influenced by their lifetime experiences, such as cognitively challenging activities or the acquisition of skills, which can act as a buffer against the negative effects of aging and diseases on the brain (Scarmeas, 2007; Stern, 2012). Measuring years of education has been considered an appropriate proxy for cognitively challenging activities and acquisition of skills. However, the ability of this relatively coarse measure to fully capture one’s ability to cope with age-related changes is debatable (Jones et al., 2011). The opportunity to pursue formal education may have been limited for certain individuals by external factors, including gender roles, race/ethnicity, childhood socioeconomic status, and geographical location. Additionally, the quality of education also differs across individuals, varying by race/ethnicity and urban/rural settings (e.g., Manly et al., 1999; Manly, 2006). Any interpretation of our findings relating years of education and cognitive aging, while consistent with previous literature, should acknowledge that the measure of formal years of education does not straightforwardly reflect one’s early-life training of contributors to crystalized and fluid intelligence or equal opportunity of access to such training.

Our study distinguishes itself from previous factor analyses of cognition in healthy aging (e.g., Adrover-Roig et al., 2012; Fisk and Sharp, 2004) by allowing tasks to load onto more than one factor. In the EFA, language tasks other than picture naming, such as Animal Fluency and sentence processing tasks, cross-loaded on both language and cognitive factors. This observation is in line with previous findings that investigated the cognitive demands of language tasks; for example, better executive functions have been associated with better performance on sentence processing tasks (Cahana-Amitay et al., 2016). As well, the hybrid nature of the animal fluency task, engaging both semantic processing and executive functions, has been extensively discussed in the literature (e.g., Shao et al., 2014; Vonk, Rizvi, et al., 2019). The Stroop task, a test that was developed to measure inhibition of automatic responses (Stroop, 1935), did not correlate with any of the four latent factors, which may indicate that the Stroop task reflects a separate cognitive ability than the rest of our testing battery. Allowing tasks to load onto more than one latent factor better represents the combined language and cognitive processes that influence performance on multicomponent tasks. These cross-loadings also support the concept of neural multifunctionality, in which neural networks for cognitive activity dynamically and continuously interact with neural networks for language abilities (Cahana-Amitay and Albert, 2014).

A limitation of our study is that cognitive and language tasks were administered at only two time points. To truly capture within-person trajectories of change over time, more follow-up measures are needed. Additionally, having only two measurement times prohibits consideration of practice effects that generally wear off after the second or third testing occasion (Vonk, Arce Rentería, et al., 2019), which may bias estimates of change over time (Vivot et al., 2016). Nonetheless, work by Mitrushina and Satz (1995) suggests that the ANT and BNT tasks may not be impacted by practice effects. Other limitations include the predominantly non-Hispanic white composition of our sample and the relatively high level of education (i.e., on average post-secondary) among our participants, which restricts generalization of these results to the general population. Moreover, during data collection, individuals’ years of education was truncated at 17 years (i.e., 17 or more years were coded as “17+”), which prevented us from analyzing education as a continuous factor. Another limitation is that a number of cognitive tasks were administered at the first evaluation but not at the second evaluation (additional tasks at first evaluation described in detail elsewhere; Higby et al., 2019), preventing longitudinal analyses of these domains. Future studies should include more cognitive measures to potentially derive more nuanced factors of cognition in addition to working memory and cognitive speed.

Our findings support the idea that language and cognition demonstrate different age-related effects during later adulthood. The differential effects of age on language domains (semantic control and semantic memory efficiency) versus cognitive domains (working memory and cognitive speed) point out the need to carefully consider examining these domains separately when studying cognitive aging. Our results furthermore suggest that it is important to observe both cross-sectional and longitudinal data to investigate relations across language and cognitive tasks and their change over time, as these study designs complement each other. As semantic processing, including both semantic control and semantic memory efficiency, plays a key role in daily communication, future studies should explore how these abilities change over time during normal aging, as well as in clinical populations in which neurodegeneration or brain damage may affect either process in isolation.

## Supplementary Material

Supplementary data are available at *The Journals of Gerontology, Series B: Psychological Sciences and Social Sciences* online.

Supplementary Text 1. Detailed description of cognitive and language assessment.

Supplementary Text 2. Detailed description of statistical analyses.

Supplementary Table S1. Distribution of variables at baseline among participants without and with missing education values, and among participants who returned for follow-up versus those who did not.

Supplementary Table S2. Correlation matrix of language and cognitive tasks (n = 306) and mean performance per task.

Supplementary Table S3. Factor loadings, effects on latent variables, and covariances in final confirmatory factor analysis model.

gbaa080_suppl_Supplementary_Material

## Funding

This work was supported by the National Institutes of Health (R01-AG014345, Drs. M. L. Albert and L. K. Obler, Co-PIs). Dr. J. M. J. Vonk was supported by an Alzheimer Nederland Fellowship (WE.15-2018-05). Dr. A. Spiro III was supported by a Senior Research Career Scientist award from the Clinical Science R&D Service, U.S. Department of Veterans Affairs.

## Conflict of Interest

None declared.
