# Supplementary material for: Demographic Effects on Longitudinal Semantic Processing, Working Memory, and Cognitive Speed
Source: J Gerontol B Psychol Sci Soc Sci. 2020 Jul 1;75(9):1850–62. doi: 10.1093/geronb/gbaa080 (PMC7759739; doi:10.1093/geronb/gbaa080)
Supplement: gbaa080_suppl_Supplementary_Material [file gbaa080_suppl_supplementary_material.docx]

## **Supplementary material**

**Supplementary Text 1.** Detailed description of cognitive and language assessments

Confrontation naming, i.e., lexical-semantic retrieval, for nouns and verbs was assessed using picture naming tasks for objects and actions using the 60-item BNT and 55-item ANT, respectively. Participants were asked to name aloud the object or action displayed in each picture. Stimuli were presented one at a time on a computer screen using E-prime software (Schneider, Eschman, & Zuccolotto, 2002). Accuracy was calculated as the percent of correct (target) responses given before any cues, and response times (in ms to the first vocalization) were measured by a voice-triggered microphone. Outliers (i.e., times exceeding 20 seconds were excluded first, and then responses > 2.5 SD from the individual’s mean), incorrect answers, and answers preceded by a cough or other vocalization were discarded from response time analyses.

Sentence comprehension was evaluated using two auditorily-presented tasks. In the Embedded

Sentences Task, participants were asked to judge the plausibility of sentences with no relative clause, with a subject-relative clause (such as “The firefighter that rescued the toddler broke a window”), or with an object-relative clause (such as “The toddler that the firefighter rescued broke a window”) (based on King & Just, 1991; Stine-Morrow, Ryan, & Leonard, 2000). The Multiple Negatives Task consisted of sentences with zero, one, or two negative markers (e.g., a sentence with two negatives might be “Because the ceiling light is not off, the room is not dark”) (described in detail in Cahana-Amitay et al., 2015; Cahana-Amitay et al., 2016; Goral et al., 2011). Both tasks were presented via E-Prime, and participants were instructed to judge whether the sentence was plausible or implausible via a response box. Accuracy was measured as percent correct. Response time data for these tasks were not analyzed because participants were not allowed to respond until after the completion of the sentence.

Verbal fluency tasks included both letter (F, A, and S) and category (animals) fluency tasks. Participants had to produce as many appropriate words as possible within 60 seconds starting with a given letter or within a given semantic category. The final score for Letter Fluency and Animal Fluency was the total number of correct items without repetitions across trials.

Working memory was assessed using Month Ordering and Digit Ordering tasks (MacDonald, Almor, Henderson, Kempler, & Andersen, 2001). Participants were read a list of months or numbers (1-19) in random order, which they had to recall in the correct order (chronologically or numerically, respectively). List length increased at each level, and their final score was the highest level of correctly recalled lists.

Shifting ability was evaluated via the Trail-making Test (Spreen & Strauss, 1998), in which participants had to draw lines to connect numbers (Trails A) and then alternating numbers and letters (1, A, 2, B, 3, C, etc.; Trails B). The time to complete Trails A and Trails B was recorded, and the Shifting score was calculated as the time difference (in seconds) between Trails A and B. Smaller difference scores indicated better shifting performance.

Inhibition was measured with the Stroop Test (Stroop, 1935). This test consisted of a list of color words printed in a color different from their meaning (e.g., the word *red* in blue letters). In the first condition, participants read aloud the words, and in the second condition, they named the ink color the words were printed in. In each condition, participants were given two minutes to read as many words or name as many colors as they could, returning to the top of the list if they reached the end before the time was up. Inhibition was measured as the difference in the number of correct items between the two conditions. Smaller difference scores indicated better performance.

Cognitive speed was calculated through Letter Comparison and Pattern Comparison tasks (Schaie, 1985). Participants were presented with a list of items, each being two strings of letters or two patterns of lines separated by a small white space between them. The task was to indicate whether those two strings or patterns were the same or different with the goal being to judge as many strings/patterns as possible within 30 seconds. Cognitive speed was assessed as the number of correctly judged items across two trials each lasting 30 seconds.

**Supplementary Text 2.** Detailed description of statistical analyses

We performed multiple imputation using Fully Conditional Specification with 10 imputations and 20 iterations to account for missing data on education; values were imputed with predictive mean matching to avoid linearity assumptions regarding imputation. The pooled estimates of the imputed values for years of education were subsequently categorized into three levels of education based on the highest educational attainment: high-school graduation or less, some or all college, or beyond college graduation (masters/doctoral degree).

The distributions of percent accuracy on the ANT, BNT, Embedded Sentences Task, and Multiple Negatives Task were left-skewed at both time points. We converted these data by subtracting the scores from the maximum percentage (100) and subsequently calculated the square root, which resulted in approximately normal distributions. Distributions on the Trails task were right-skewed at both time points. A logarithmic transformation resulted in approximately normal distributions for these data. Performance on the other cognitive and language tasks was approximately normally distributed at both time points. For ease of interpretation so that on every test a higher score reflected better performance, scores on ANT accuracy, ANT response time, BNT accuracy, BNT response time, Embedded Sentences Task, Multiple Negatives Task, and Trails were mirrored through multiplication by -1.

To model the underlying factor structure of the cognitive and language tasks, we performed an Exploratory Factor Analysis (EFA) with oblique rotation, which allows for correlation among factors, and a maximum likelihood estimation method. We included the 14 cognitive and language tasks at baseline with standardized scores in models with up to 7 factors. An eigenvalue analysis, including a scree plot, was performed to get an estimate for the number of underlying dimensions. To assess goodness of fit of the EFA models, we used the following guidelines: Chi-square goodness of fit test p > .05, Root Mean Square Error of Approximation (RMSEA) < .05, Comparative Fit Index (CFI) > 0.90, Tucker Lewis Index (TLI) > 0.90, and Standardized Root Mean Squared Residual (SRMR) < 0.07 (Yu, 2002). To compare models with a different number of factors, we compared the Akaike Information Criterion (AIC) values, in which the smaller of two values indicates a better fit. Item loadings with values of .25 or larger were considered for each factor.

We performed Confirmatory Factor Analysis (CFA) to investigate if the effects of age and education were different among the latent factors identified by the EFA. To avoid convergence issues that may result from large differences in the variances because each task was measured on its own scale, we standardized all measures. Missingness in the EFA and CFA was accounted for by the Full Information Maximum Likelihood (FIML) method. Values were missing at baseline for 12.1% of the scores on ANT accuracy, 14.1% on ANT response time, 4.9% on BNT accuracy, 6.2% on BNT response time, 11.8% on the Embedded Sentences Task, 18.0% on the Multiple Negatives Task, 22.5% on Letter Fluency, 23.2% on Animal Fluency, 10.8% on Month Ordering, 12.1% on Digit Ordering, 28.8% on Letter Comparison, 29.1% on Pattern Comparison, 8.8% on Stroop, and 12.1% on Trails.

The CFA model included the identified factors from the EFA as latent variables based on the observed cognitive and language tasks at baseline, with age, education, and sex regressed on the latent variables. After model specification, model fit was assessed using RMSEA, CFI, TLI, and SRMR. Modification indices combined with conceptual judgment were used for model improvement, and model improvement was assessed by AIC values. The resulting model was used for hypothesis testing. To compare the estimates of the effects of age and education on the latent factor parameters, linear restrictions on the parameters in the model were tested using the Wald chi-square test.

Change over time in the latent factors defined in the CFA was investigated using linear mixed models. To preserve within-subject change over time, we estimated factor scores from the CFA for each participant at both time points by multiplication of standardized test scores by the CFA loadings, without adjustment for demographic effects; an individual’s latent factor value was only calculated if at least two tasks that contributed to the factor were administered. To ensure that the internal structure of our measurement battery was equal across time (i.e., measurement invariance), we tested for configural, metric, and scalar invariance with methods described in detail in Avila et al. (2019).

Visual inspection of the shape of the relationship between each latent factor and age was performed by fitting a smoothed curve computed by loess on the observed data. If the shape of the smoothed curve suggested a non-linear relationship, a basis spline was fitted to perform piecewise linear modeling within the mixed model. AICs of models without and with a spline were compared, and optimal placement of knots was assessed by comparing models’ AIC.

Mixed models included one of the latent factors as the outcome. Time in the study parameterized by age (from baseline age to follow-up age, which accounts for individually-varying follow-up intervals), as well as educational attainment and sex, were included as fixed factors, together with a random intercept and random slope. Subsequent models additionally included the interaction between time in study (parameterized by age) and educational attainment to test for moderation by education on slope.

**Supplementary Text 3.** Establishing measurement invariance

To ensure that the internal structure of our measurement battery was equal across time (i.e., measurement invariance), we tested for configural, metric, and scalar invariance with methods described in detail in Avila et al. (2019) in the CFA model without adjustment for demographic effects.

Across time, the configural model fit well (χ2 (246)=366.944, p<.001; RMSEA=.040, CFI=.932, TLI=.910, SRMR=.065, AIC=11741.846). Examination of goodness of fit indices indicated that the data did not fit full metric invariance (χ^2^ (261)=410.228, p<.001; RMSEA=.043, CFI=.916, TLI=.895, SRMR=.075, AIC=11755.131), but did reach partial metric invariance after releasing the constraints across factor loadings of animal fluency, Multiple Negatives Task, and Trails (χ^2^ (255)=382.287, p<.001; RMSEA=.040, CFI=.928, TLI=.908, SRMR=.066., AIC=11739.189). Based on the partial metric model, goodness of fit indices indicated that the data fit scalar invariance (χ^2^ (264)=382.764, p<.001; RMSEA=.038, CFI=.933, TLI=.917, SRMR=.067, AIC=11721.666). Additionally, we assessed model fit of the final CFA model with demographic effects on the follow-up measurements: RMSEA=.069, CFI=.910, TLI=.868, and SRMR=.065.

**Supplementary Table S1.** Distribution of variables at baseline among participants without and with missing education values, and among participants who returned for follow-up versus those who did not

|  | Not missing  education (n = 292) | Missing  education (n = 14) | Returned for follow-up (n = 116) | Not returned for follow-up (n = 190) |
| --- | --- | --- | --- | --- |
| Age at baseline (mean (SD)) | 71.68 (7.73) | 69.93 (7.04) | 71.16 (6.54) | 71.87 (8.33) |
| Sex/gender (n, % women) | 145 (49.7) | 5 (35.7) | 61 (52.6) | 89 (46.8) |
| Race/Ethnicity (n, %) |  |  |  |  |
| Non-Hispanic White | 243 (85.3) | 3 (50.0) | 99 (90.8) | 147 (80.8) |
| Non-Hispanic Black | 37 (13.0) | 3 (50.0) | 7 (6.4) | 33 (18.1) |
| Hispanic | 2 (0.7) | 0 (0.0) | 1 (0.9) | 1 (0.5) |
| Other | 3 (1.1) | 0 (0.0) | 2 (1.8) | 1 (0.5) |
| MMSE | 28.93 (1.18) | 28.00 (1.79) | 28.83 (1.29) | 29.01 (1.05) |
| ANT accuracy (mean (SD)) | 95.98 (3.92) | 95.98 (2.42) | 96.13 (4.00) | 95.87 (3.80) |
| ANT RT (mean (SD)) | 1350.22 (310.43) | 1482.61 (293.05) | 1339.46 (288.01) | 1364.17 (325.97) |
| BNT accuracy (mean (SD)) | 92.61 (6.88) | 92.94 (5.13) | 93.02 (6.95) | 92.36 (6.76) |
| BNT RT (mean (SD)) | 1290.58 (271.35) | 1470.40 (284.80) | 1277.77 (258.00) | 1308.02 (282.38) |
| Embedded Sentences Task (mean (SD)) | 89.16 (8.68) | 89.58 (6.84) | 89.37 (10.08) | 89.04 (7.49) |
| Multiple Negatives Task (mean (SD)) | 92.69 (6.08) | 88.67 (10.48) | 92.80 (5.85) | 92.44 (6.50) |
| Letter Fluency (mean (SD)) | 45.71 (13.56) | 49.67 (19.86) | 46.30 (13.82) | 45.40 (13.49) |
| Animal Fluency (mean (SD)) | 17.51 (5.25) | 20.67 (3.51) | 18.19 (5.27) | 17.12 (5.20) |
| Month Ordering span (mean (SD)) | 4.28 (0.99) | 3.58 (1.02) | 4.23 (0.95) | 4.29 (1.03) |
| Digit Ordering span (mean (SD)) | 4.61 (0.87) | 4.50 (0.52) | 4.74 (0.89) | 4.51 (0.84) |
| Stroop difference score (mean (SD)) | 147.46 (40.28) | 165.00 (54.82) | 151.41 (39.47) | 145.61 (41.38) |
| Trails difference score (mean (SD)) | 46.98 (30.20) | 54.50 (25.18) | 44.32 (24.50) | 49.26 (33.37) |
| Letter Comparison (mean (SD)) | 17.15 (4.26) | 14.62 (2.39) | 17.68 (4.20) | 16.63 (4.21) |
| Pattern Comparison (mean (SD)) | 28.78 (5.93) | 25.75 (5.42) | 29.09 (6.38) | 28.37 (5.60) |

*Note.* SD = standard deviation; ANT = Action Naming Test; BNT = Boston Naming Test; RT = response time; MMSE = Mini Mental State Exam

**Supplementary Table S2.** Correlation matrix of language and cognitive tasks (n = 306) and mean performance per task

|  | ANT acc | ANT RT | BNT acc | BNT RT | RCJ | MNJ | Letter-F | Animal-F | MO | DO | Stroop | Trails | LC | PC |
| --- | --- | --- | --- | --- | --- | --- | --- | --- | --- | --- | --- | --- | --- | --- |
| ANT acc | 1.000 |  |  |  |  |  |  |  |  |  |  |  |  |  |
| ANT RT | .323 | 1.000 |  |  |  |  |  |  |  |  |  |  |  |  |
| BNT acc | .544 | .392 | 1.000 |  |  |  |  |  |  |  |  |  |  |  |
| BNT RT | .371 | .633 | .417 | 1.000 |  |  |  |  |  |  |  |  |  |  |
| EST | .343 | .104 | .298 | .160 | 1.000 |  |  |  |  |  |  |  |  |  |
| MNJ | .210 | .087 | .199 | .077 | .366 | 1.000 |  |  |  |  |  |  |  |  |
| Letter-F | .297 | .169 | .272 | .281 | .299 | .198 | 1.000 |  |  |  |  |  |  |  |
| Animal-F | .280 | .320 | .355 | .441 | .362 | .216 | .337 | 1.000 |  |  |  |  |  |  |
| MO | .217 | .100 | .202 | .158 | .360 | .346 | .306 | .371 | 1.000 |  |  |  |  |  |
| DO | .178 | .067 | .171 | .193 | .192 | .211 | .254 | .218 | .511 | 1.000 |  |  |  |  |
| Stroop | .012 | .020 | .083 | -.039 | .138 | .035 | -.117 | -.015 | -.004 | -.095 | 1.000 |  |  |  |
| Trails | .318 | .251 | .300 | .271 | .257 | .234 | .358 | .332 | .366 | .291 | -.027 | 1.000 |  |  |
| LC | .240 | .299 | .178 | .348 | .320 | .175 | .412 | .301 | .319 | .282 | -.024 | .392 | 1.000 |  |
| PC | .207 | .340 | .161 | .316 | .263 | .086 | .244 | .344 | .190 | .128 | .036 | .302 | .600 | 1.000 |
| *Mean*  *SD* | 95.98 (3.88) | 1353.74 (310.18) | 92.62 (6.83) | 1296.22 (273.07) | 89.18 (8.62) | 92.60 (6.22) | 45.76 (13.60) | 17.55 (5.24) | 4.27 (0.99) | 4.61 (0.87) | 147.90 (40.67) | 47.20 (30.05) | 17.06 (4.23) | 28.66 (5.93) |

*Note.* Acc = accuracy, RT = response time, ANT = Action Naming Test, BNT = Boston Naming Test, EST = Embedded Sentences Task, MNT = Multiple Negatives Task, Letter-F = Letter Fluency, Animal-F = Animal Fluency, MO = Month Ordering span, DO = Digit Ordering span, LC = Letter Comparison, PC = Pattern Comparison, SD = standard deviation

**Supplementary Table S3.** Factor loadings, effects on latent variables, and covariances in final confirmatory factor analysis model

|  |  | Estimate | SE | p-value |
| --- | --- | --- | --- | --- |
| *Factor loadings* | |  |  |  |
| Semantic control | ANT accuracy | 1.000 | .000 | - |
|  | BNT accuracy | 1.183 | .131 | .000 |
|  | Embedded Sentences Task | -.092 | .190 | .630 |
|  | Multiple Negatives Task | -.204 | .183 | .265 |
| Semantic memory | ANT RT | 1.000 | .000 | - |
|  | BNT RT | 1.156 | .129 | .000 |
|  | Animal fluency | .417 | .109 | .000 |
| Working memory | Embedded Sentences Task | 1.000 | .000 | - |
|  | Multiple Negatives Task | 1.205 | .243 | .000 |
|  | Animal fluency | .778 | .194 | .000 |
|  | Letter fluency | .610 | .201 | .002 |
|  | Month ordering span | .940 | .216 | .000 |
|  | Digit ordering span | .590 | .160 | .000 |
|  | Trails difference score | .591 | .186 | .001 |
| Cognitive speed | Letter fluency | 1.000 | .000 | - |
|  | Trails difference score | 1.088 | .531 | .041 |
|  | Letter comparison | 3.404 | 1.463 | .020 |
|  | Pattern comparison | 3.131 | 1.455 | .031 |
| *Demographic effects on the latent variables* | | |  |  |
| Age | Semantic control | -.012 | .006 | .042 |
|  | Semantic memory | -.007 | .007 | .304 |
|  | Working memory | -.022 | .006 | .001 |
|  | Cognitive speed | -.011 | .005 | .020 |
| Education | Semantic control | .342 | .069 | .000 |
|  | Semantic memory | .208 | .071 | .003 |
|  | Working memory | .252 | .078 | .001 |
|  | Cognitive speed | .064 | .039 | .104 |
| Sex/gender | Semantic control | -.318 | .089 | .000 |
|  | Semantic memory | -.113 | .097 | .243 |
|  | Working memory | .155 | .084 | .064 |
|  | Cognitive speed | .065 | .047 | .168 |
| *Covariances* | |  |  |  |
| Semantic memory with semantic control | | .261 | .049 | .000 |
| Working memory with semantic control | | .205 | .065 | .002 |
| Working memory with semantic memory | | .116 | .048 | .016 |
| Cognitive speed with semantic control | | .043 | .025 | .085 |
| Cognitive speed with semantic memory | | .084 | .041 | .039 |
| Cognitive speed with working memory | | .047 | .024 | .050 |
| Digit ordering with month ordering | | .314 | .060 | .000 |

SE = standard error; ANT = Action Naming Test; BNT = Boston Naming Test; RT = response time

REFERENCES SUPPLEMENTARY MATERIAL

Avila, J. F., Arce Rentería, M., Witkiewitz, K., Verney, S. P., Vonk, J. M., & Manly, J. J. (2019). Measurement invariance of neuropsychological measures of cognitive aging across race/ethnicity by sex/gender groups. *Neuropsychology, 34*(1), 3-14. doi:10.1037/neu0000584

Cahana-Amitay, D., Spiro III, A., Cohen, J. A., Oveis, A. C., Ojo, E. A., Sayers, J. T., . . . Albert, M. L. (2015). Effects of metabolic syndrome on language functions in aging. *Journal of the International Neuropsychological Society, 21*(2), 116-125. doi:10.1017/S1355617715000028

Cahana-Amitay, D., Spiro III, A., Sayers, J. T., Oveis, A. C., Higby, E., Ojo, E. A., . . . Albert, M. L. (2016). How older adults use cognition in sentence-final word recognition. *Aging, Neuropsychology, and Cognition, 23*(4), 418-444. doi:10.1080/13825585.2015.1111291

Goral, M., Clark-Cotton, M., Spiro III, A., Obler, L. K., Verkuilen, J., & Albert, M. L. (2011). The contribution of set switching and working memory to sentence processing in older adults. *Experimental Aging Research, 37*(5), 516-538. doi:10.1080/0361073X.2011.619858

King, J., & Just, M. A. (1991). Individual differences in syntactic processing: The role of working memory. *Journal of Memory and Language, 30*(5), 580-602. doi:10.1016/0749-596X(91)90027-H

MacDonald, M. C., Almor, A., Henderson, V. W., Kempler, D., & Andersen, E. S. (2001). Assessing working memory and language comprehension in Alzheimer's disease. *Brain and Language, 78*(1), 17-42. doi:10.1006/brln.2000.2436

Schaie, K. W. (1985). *Manual for the Schaie-Thurstone adult mental abilities test (STAMAT)*. Palo Alto, CA: Consulting Psychologists Press.

Schneider, W., Eschman, A., & Zuccolotto, A. (2002). *E-Prime User's Guide*. Pittsburgh, PA: Psychology Software Tools, Inc.

Spreen, O., & Strauss, E. (1998). *A Compendium of Neuropsychological Tests: Administration, Norms, and Commentary*. New York: Oxford Univeristy Press.

Stine-Morrow, E. A. L., Ryan, S., & Leonard, J. S. (2000). Age differences in on-line syntactic processing. *Experimental Aging Research, 26*(4), 315-322. doi:10.1080/036107300750015714

Stroop, J. R. (1935). Studies of interference in serial verbal reactions. *Journal of Experimental Psychology, 18*(6), 643-662. doi:10.1037/h0054651

Yu, C.-Y. (2002). *Evaluating cutoff criteria of model fit indices for latent variable models with binary and continuous outcomes*. Los Angeles, CA: University of California, Los Angeles.
